# Mortality in women of reproductive age in rural South Africa

**DOI:** 10.3402/gha.v6i0.22834

**Published:** 2013-12-19

**Authors:** Dorean Nabukalu, Kerstin Klipstein-Grobusch, Kobus Herbst, Marie-Louise Newell

**Affiliations:** 1 Division of Epidemiology and Biostatistics, School of Public Health, Faculty of Health Sciences, University of the Witwatersrand, Johannesburg, South Africa; 2 Rakai Health Sciences Program, Uganda Virus Research Institute, Entebbe, Uganda; 3 Julius Global Health, Julius Center for Health Sciences and Primary Care, University Medical Center Utrecht, Utrecht, The Netherlands; 4 Africa Centre for Health and Population Studies, University of KwaZulu-Natal, Somkhele, South Africa; 5 Faculty of Medicine and Faculty of Social and Human Sciences, University of Southampton, Southampton, UK

**Keywords:** reproductive age, women, mortality, rural South Africa, risk factors

## Abstract

**Objective:**

To determine causes of death and associated risk factors in women of reproductive age in rural South Africa.

**Methods:**

Deaths and person-years of observation (pyo) were determined for females (aged 15–49 years) resident in 15,526 households in a rural South African Demographic and Health Surveillance site from 2000 to 2009. Cause of death was ascertained by verbal autopsy and ICD-10 coded; causes were categorized as HIV/TB, non-communicable, communicable/maternal/perinatal/nutrition, injuries, and undetermined (unknown). Characteristics of women were obtained from regularly updated household visits, while HIV and self-reported health status was obtained from the annual HIV surveillance. Overall and cause-specific mortality rates (MRs) with 95% confidence intervals (CI) were calculated. The Weibull regression model (HR, 95% CI) was used to determine risk factors associated with mortality.

**Results:**

A total of 42,703 eligible women were included; 3,098 deaths were reported for 212,607 pyo. Overall MRwas 14.6 deaths/1,000 pyo (95% CI: 14.1–15.1), peaking in 2003 (MR 18.2/1,000 pyo, 95% CI: 16.4–20.1) and declining thereafter (2009: MR 9.6/1,000 pyo, 95% CI: 8.4–10.9). Mortality was highest for HIV/TB (MR 10.6/1,000 pyo, 95% CI: 10.2–11.1), accounting for 73.1% of all deaths, ranging from 61.2% in 2009 to 82.7% in 2002. Adjusting for education level, marital status, age, employment status, area of residence, and migration, all-cause mortality was associated with external migration (adjusted hazard ratio, or aHR), 1.70, 95% CI: 1.41–2.05), self-reported poor health status (aHR 8.26, 95% CI: 2.94–23.15), and HIV-infection (aHR 7.84, 95% CI: 6.26–9.82); external migration and HIV infection were also associated with causes of mortality other than HIV/TB (aHR 1.62, 95% CI: 1.12–2.34 and aHR 2.59, 95% CI: 1.79–3.75).

**Conclusion:**

HIV/TB was the leading cause of death among women of reproductive age, although rates declined with the rollout of HIV treatment in the area from 2004. Women's age, external migration status and HIV-positive status were significantly associated with all-cause and cause-specific mortality.

In many settings, women are confronted by a dual burden of traditional health threats related to infectious diseases and maternal conditions alongside emerging challenges associated with non-communicable chronic diseases (NCD). Consequently, more than 30% of all deaths in resource-limited settings occur in women aged 15–49 years, while in resource-rich settings this is 15%. Maternal mortality is the lead cause of mortality in women of this age group in sub-Saharan Africa, and NCDs are the major cause of death in resource-rich countries (1, 2).

Since maternal mortality is an important problem in sub-Saharan Africa (where fertility is also high with limited or no skilled birth-attended deliveries and poor antenatal clinic, or ANC, attendance), a number of studies responding to the Millennium Development Goals have been conducted to investigate maternal mortality and associated risk factors which include old age, poor ANC attendance, belonging to a lower socio economic class, low education level, distance to a health facility, and being resident in a rural area (3–7). Studies carried out in this part of rural South Africa have documented low fertility rates, high ANC attendance, high HIV incidence and prevalence, and high poverty levels in women of reproductive ages (8–10). Therefore, in this study, we look at mortality generally in all women of reproductive age and clarify causes of death and associated risk factors.

In resource-limited countries, deaths are often under-reported, and cause of death is frequently not available due to lack of vital registration systems (11, 12). Such a lack of data hinders the understanding of risk factors for different causes of deaths, although HIV-related deaths have recently been studied in more detail, often within specific studies rather than from vital registration. To improve national vital registration, a number of countries intend to set up a nationally representative vital registration system sample, which has been recommended to represent an affordable, cost-effective, and sustainable short- and medium-term solution if applied in conjunction with validated verbal autopsy (VA) procedures as has been demonstrated by work conducted in Health and Demographic Surveillance Sites (13, 14).

This study uses longitudinal data from a large ongoing surveillance study in rural South Africa, a setting with high HIV prevalence (27% in women) and incidence (3.2 per 100 person-years) in women aged 15–49 years (8, 15), and quantifies mortality in women of reproductive age (aged 15–49 years).

## Methods

The Africa Centre for Health and Population Studies is located near the market town of Mtubatuba in the uMkhanyakude district of KwaZulu-Natal. The surveillance area covers an area of 438 km^2^, and within each surveillance round a population of approximately 90,000 people, of whom about two-thirds are resident in approximately 11,000 households (8). The population is almost exclusively Zulu-speaking and the area is typical of many rural areas of South Africa in that while predominantly rural, it contains an urban township and informal peri-urban settlements (8, 16). From 2000 to 2011, the Centre hosted a biannual (tri-annual since 2012) sociodemographic surveillance, in which all households in the area were visited and demographic indicators recorded, including household size and composition, fertility, migration, mortality, and marriage. The Africa Centre collects information on both resident and non-resident household members and makes a distinction between membership and residency. Household membership is self-defined on the basis of links to other household members while residency implies residing at a physical structure within the surveillance area at a particular point in time (8). In addition, since 2003, adults aged 15 years and over, identified as residents in the household surveillance, are asked to participate in an annual HIV and health surveillance (17). More information about the Africa Centre operations and profile is available elsewhere (8, 17, 18).

The study sample constituted an open cohort of all women aged 15–49 years who were members of households within the demographic surveillance area (DSA) during the period 2000–2009, a period for which complete data was available at the time of analysis. All resident membership episodes of the females aged 15–49 years at any time during the period 2000–2009 were included. Household membership is as reported by the key household informant. It starts with a demographic surveillance system (DSS) entry, a birth or a membership start event and can only be terminated by DSS exit, a death or a membership end event. Non-resident episodes of members who exited and then returned were taken into account but person time was only computed for the period they were resident.

### Mortality data

Although all deaths (of both resident and non-resident household members) reported in the household surveillance are followed by a VA interview with the closest caregiver of the deceased, for this study we looked at residents for causes of mortality since only resident episodes are taken into account while computing person time and thus mortality rates (MRs). VA is an epidemiological tool that is used to assess cause of death in settings where hospital-based records are lacking or inadequate. During the period of this study, a VA interview was conducted on average 9 months after a death was reported (17, 19). In interviews conducted by a trained nurse, informants were asked to narrate the course of the illness or events leading to death. A structured interview was also administered. Two experienced clinicians independently assigned the underlying cause of death^1^ based on the information collected in the VA and their clinical judgment using the 10th version of the International Classification of Diseases (ICD-10) coding system (17, 19–22). Validation of VA as a tool for assigning cause of death has been documented by earlier studies (11, 12, 23). Following the Global Burden of Disease classification, causes were divided into three broad categories: communicable, maternal, perinatal, and nutritional conditions (group I); non-communicable diseases (group II); and group III which contained injuries (24). For the purposes of this study, group I causes of death were further categorized into HIV/TB-related, maternal, and other communicable causes. HIV/TB-related causes were grouped together because of the considerable overlap in mortality from HIV infection and tuberculosis (25). Data – on age, education, pregnancy status and outcome, marital, and migration status – were collected during the household visits using a structured questionnaire administered to the key household informant (8). Information on self-reported health and contraceptive use were collected during the annual individual surveillance using the ‘Woman's General Health’ (WGH) questionnaire (18).

Household socioeconomic data (HSE) were collected once a year within the household surveillance, collecting information from the key informant on housing structure, sources of energy and amenities, education and employment of household members (18).

HIV status data is updated once a year from the HIV surveillance visits for participating adults. After written informed consent, the field workers collect blood by finger prick and prepare dried blood spots for ano-nymized HIV testing (8). HIV status is determined by antibody testing with a broad-based HIV-1/HIV-2 enzyme-linked immunosorbent assay (ELISA; Vironostika, Organon Teknika, Boxtel, the Netherlands) followed by a confirmatory ELISA (GAC-ELISA; Abbott, Abbott Park, Illinois, USA).

The risk factors, which varied over time, were fixed and measured at particular points in time. These factors include age of the woman at the birth of her most recent child (5, 7, 12, 19, 21, 26), migration status (20, 27, 28) (defined as: never moved, internal migration (within the surveillance area) only, external migration (migration in/out of the surveillance area) only, and both internal and external migration), HIV status (21, 29, 30) (defined as: participated in the HIV surveillance and positive, participated in the HIV surveillance and negative, and unknown status for those who never participated in the HIV surveillance), area of residence (rural, urban, and peri-urban), parity at most recent birth, pregnancy outcome summarized across maternity history (12, 31) (all live births, one or more pregnancy ended in a stillbirth, no pregnancy outcome reported), contraceptive use (ever or never use), marital status (ever married and never married), self-reported general health status (lowest self-reported health status over the period; defined as good, fair or poor), highest education level (4, 19) (categorized as eight or more years in school, less than eight years in school and no school at all), employment status (ever or never employed) and wealth index of the household where the woman lives (minimum wealth index of all households a woman has ever stayed in during the study period; the derivation of the wealth index is explained below).

As a proxy to measure household wealth, a household asset index was used (32). The household asset index is computed by principal component analysis (28) using information on house ownership, water source, energy, toilet type, electricity and ownership of assets which included items that can be used for consumption, production or both, such as beds, bicycles, tables, telephones, television sets, sewing machines, block makers, wheelbarrows, tractors, cattle, and other livestock (9, 33). Households were then categorized as either belonging to the poorest 40%, the middle 40% or the wealthiest 20% on the asset index scale (9, 33). These three categories were chosen for comparison purposes with other studies since they have been found to capture wealth effects well in a number of studies in poor provinces of South Africa (9, 34).

### Statistical analysis

Cross tabulation of categorical variables and vital status was performed with Chi-square tests of significant differences within the variable groups and a woman's vital status; p-values were reported.

Data were set for survival analysis with vital status as the failure variable. The time variable was the difference in years between entry (the date at which an individual was first observed/visited) and exit date (the date an individual was last observed/visited; i.e. for deaths the exit date was the date of death while for the surviving participants, it was the end of the study date). Participants who left the study before the end point or before 31 December 2009 (due to out-migration or loss to follow-up) were censored on the dates last observed, and the person-years they contributed were computed up to their last observation dates. Percentage distributions of each of the different causes of death to the overall number of deaths were computed. MRs for the different causes of death and the overall MR were computed per 1,000 women years of observation (pyo). Causes of death were divided into two categories: deaths due to HIV/TB and deaths due to all other causes combined, because there were small numbers reported for deaths from other causes (35). A Cox regression model was fitted to test for association between factors and mortality over time. Scaled Schoenfield residuals were used to test the final model for violation of the assumption of proportionality over the study time (2000–2009). HIV status, self-reported minimum health status, employment status, and woman's age at birth of most recent child violated the proportionality assumptions, the model was stratified by these variables but the assumptions remained violated. Thus, a parametric model (the Weibull distribution) was used (35) to assess factors associated with all-cause mortality and cause-specific mortality at 5% level of significance. The Weibull model was chosen over other parametric models because of its flexibility and ability to model hazard functions that are decreasing, increasing or constant over time. The model was explored for interaction effects and age was found to modify the effect of general health on all-cause mortality as well as cause-specific mortality. This interaction term was included in the models and adjusted results are reported in 3–5. All analyses were computed using STATA v 11 (36).

This study was approved by the University of Witwatersrand Committee for Research on Human Subjects and ethical approval for the Africa Centre Surveillance was obtained from the Research Ethics Committee of the Nelson R. Mandela School of Medicine, University of KwaZulu-Natal.

## Results

A total of 42,703 women were enrolled, of whom 3,098 died during the period, with a total of 212,607 person-years of observation. Most deaths occurred in women aged 25–29 years (21.1%) and most participants lived in a rural setting (Table 1). Although migration was common among women who were alive when last seen, most women who died (42.41%) had never moved either within (internally) or out of/into (externally) the surveillance area (Table 1). While most of the women had completed 8 or more years in school, 31.4% of those who died had completed less than 8 years of education. There were significant differences between women who had died and those who were alive when last seen across the three categories of HIV status (Table 1).

**Table 1:** Sociodemographic characteristics of women of reproductive age during the study period, ACDIS

Variable	Women alive (0) (n)%	Women dead (1) (n)%	P-value
Overall (n = 42,703)	(n = 39,605) 92.75	(n = 3,098) 7.25	
Age in years at entry			<0.001
15–19	(18,220) 46.0	(491) 15.85	
20–24	(6,174) 15.59	(615) 19.85	
25–29	(4,722) 11.92	(655) 21.14	
30–34	(3,579) 9.04	(534) 17.24	
35–39	(2,895) 7.31	(402) 12.98	
40–44	(2,346) 5.92	(293) 9.46	
45–49	(1,669) 4.21	(108) 3.49	
Area of residence			<0.001
Rural	(22,737) 57.4	(1,802) 58.17	
Urban	(4,023) 10.16	(142) 4.58	
Peri-urban	(12,753) 32.20	(1,154) 37.25	
Migration status			<0.001
Never moved	(11,495) 29.02	(1,314) 42.41	
Internal only	(3,982) 10.05	(273) 8.81	
External only	(7,237) 18.27	(606) 19.56	
Internal and external	(16,891) 42.65	(905) 29.21	
Minimum HSE class index			<0.001
Upper 20%	(4,568) 12.73	(225) 7.59	
Middle 40%	(12,064) 33.61	(1,007) 33.96	
Lower 40%	(19,263) 53.66	(1,733) 58.45	
Ever married			0.015
No	(32,752) 82.7	(2,615) 84.41	
Yes	(6,853) 17.3	(483) 15.59	
Ever employed			0.353
No	(29,701) 74.99	(2,300) 74.24	
Yes	(9,904) 25.01	(798) 25.76	
Highest level of education			<0.001
More than 8 years	(24,647) 71.88	(1,469) 62.25	
Less than 8 years	(8,083) 23.57	(742) 31.44	
No school	(1,559) 4.55	(149) 6.31	
Self-reported minimum general health			<0.001
Good	(17,095) 79.68	(770) 69.43	
Fair	(3,808) 17.75	(253) 22.81	
Poor	(5,51) 2.57	(86) 7.75	
Woman HIV status			<0.001
HIV tested and negative	(9,532) 24.07	(117) 3.78	
HIV tested and positive	(4,645) 11.73	(532) 17.17	
Never tested	(25,428) 64.20	(2,449) 79.05	
Maximum parity (M, SD)	1.79, 2.13	2.28, 2.06	<0.001
Pregnancy outcome summarized from maternity history			<0.001
All live births	(22,005) 55.56	(1,917) 61.88	
≥ 1 pregnancy was a still birth	(29,68) 7.49	(317) 10.23	
No outcome reported	(14,632) 36.94	(864) 27.89	

### Causes of death

HIV/TB-related causes of death were most common with a MR of 10.64 per 1,000 person-years (95% CI: 10.21–11.08), contributing almost 73% of all deaths to women of reproductive age over the entire period (Table 2). There was a significant difference in the mortality fraction due to HIV/TB-related causes ranging from as high as 82.7% in 2002 to as low as 61.2% in 2009. Women who died while pregnant contributed 0.45% of all deaths in women of reproductive age with a MR of 0.07 per 1,000 person-years (95% CI: 0.04–0.11) and non-communicable diseases (mainly cancers) contributed 8.42% with a MR of 1.22 per 1,000 person-years (95% CI: 1.08–1.438).

**Table 2:** Causes of death in women of reproductive age, 2000–2009, ACDIS

				95% confidence intervals	
				
Causes of death	Cause-specific numbers	Mortality fraction (%)	Mortality rate /1,000 person-years	Lower	Upper
HIV/TB	2,266	73.14	10.64	10.21	11.08
*Pregnancy	14	0.45	0.07	0.04	0.11
Other communicable	266	8.59	1.25	1.11	1.41
Injuries	109	3.52	0.51	0.42	0.62
Non communicable	261	8.42	1.22	1.08	1.38
Unknown	182	5.87	0.86	0.74	0.99

* This refers to deaths that happened to women while they were pregnant.

### Mortality patterns

**Fig. 1:**
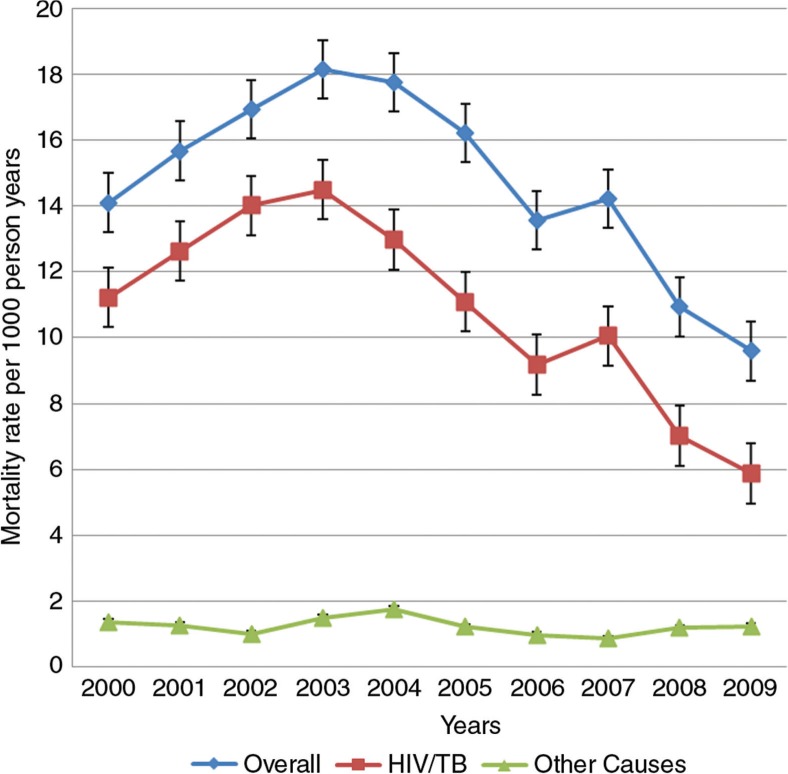
All-cause and cause-specific mortality rates for women of reproductive age from 2000 to 2009, ACDIS.

**Fig. 2:**
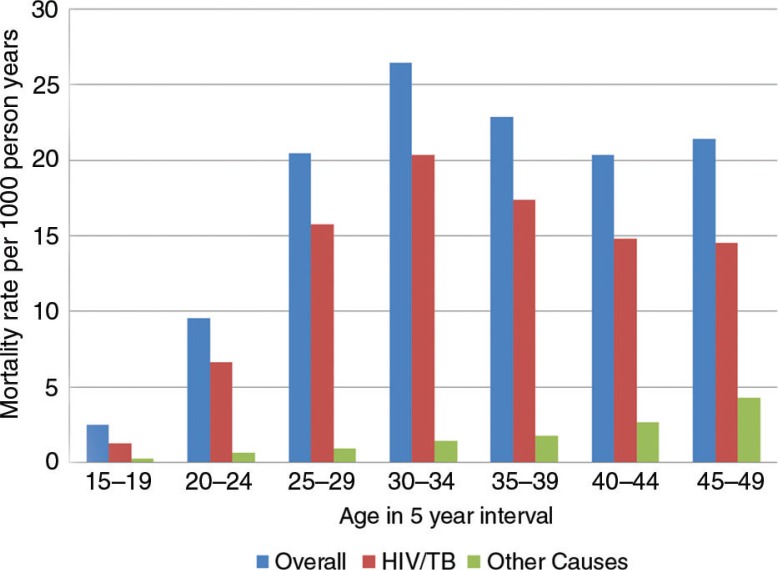
All-cause and cause-specific mortality rates by age group from 2000 to 2009, ACDIS.

The all-cause crude MR for women of reproductive age was 14.6 per 1,000 person-years (95% CI: 14.1–15.1), varying from 9.6 (in 2009) per 1,000 person-years to 18.2 (in 2003) per 1,000 pyo (95% CI: 16.4–20.1) (Fig. 1). The overall pattern was similar to that seen for HIV/TB-related causes only, but differed significantly for other causes. All-cause crude MRs were highest in women 30–34 years old, while MRs due to causes other than HIV/TB-related were highest for women aged 45–49 years (Fig. 2).

### Risk factors of mortality

Increasing age was significantly associated with both all-cause and cause-specific mortality, in the unadjusted model and after adjusting for all other sociodemographic indicators. The risk of all-cause mortality and of mortality related to HIV/TB was highest for women aged 30–34 years; for mortality due to causes not related to HIV/TB, the risk was highest for women aged 25–29 years (Tables (3–5)). For all-cause mortality and mortality due to HIV/TB, the hazards of dying were almost four- and five-times increased in women aged 30–34 years compared to the reference group of those aged 15–19 years, keeping all other sociodemographic factors constant.

**Table 3:** Univariable and multivariable hazard ratios (95% CI) for risk factors associated with all-cause mortality, 2000–2009, ACDIS

		Univariable analysis			Multivariable analysis		
			
Explanatory variables	Categories of explanatory variables	Crude hazards ratio	95% CI	P value	Adjusted hazards ratio	95% CI	P value
Women age	15–19	1.000					
	20–24	4.267	3.506, 5.194	<0.001	2.186	1.435, 3.330	<0.001
	25–29	9.336	7.742, 11.257	<0.001	3.457	1.980, 6.036	<0.001
	30–34	11.910	9.873, 14.367	<0.001	3.615	1.726, 7.573	0.001
	35–39	10.309	8.491, 12.516	<0.001	2.857	1.123, 7.269	0.028
	40–44	9.205	7.541, 11.237	<0.001	1.528	0.489, 4.775	0.465
	45–49	9.908	8.083, 12.145	<0.001	1.563	0.411, 5.946	0.512
Self-reported minimum general health	Good	1.000			1.000		
	Fair	1.293	1.122, 1.491	<0.001	2.302	1.215, 4.358	0.010
	Poor	2.927	2.343, 3.658	<0.001	8.256	2.944, 23.151	<0.001
Ever employed	No	1.000			1.000		
	Yes	0.622	0.574, 0.675	<0.001	0.779	0.681, 0.891	<0.001
Ever married	No	1.000			1.000		
	Yes	0.665	0.604, 0.733	<0.001	0.500	0.419, 0.598	<0.001
Migration status	Never moved	1.000			1.000		
	Internal only	0.451	0.396, 0.514	<0.001	0.647	0.527, 0.796	<0.001
	External only	1.549	1.405, 1.709	<0.001	1.698	1.408, 2.046	<0.001
	Both internal and external	0.377	0.346, 0.410	<0.001	0.476	0.406, 0.557	<0.001
Minimum household index class	Upper 20%	1.000			1.000		
	Middle 40%	1.505	1.302, 1.739	<0.001	1.077	0.809, 1.433	0.611
	Lower 40%	1.527	1.329, 1.754	<0.001	1.167	0.872, 1.563	0.298
Highest education level	More than 8 years	1.000			1.000		
	Less than 8 years	1.855	1.698, 2.026	<0.001	1.338	1.139, 1.572	<0.001
	Never gone to school	1.618	1.367, 1.915	<0.001	1.334	1.023, 1.739	0.033
Ever used contraception	No	1.000			1.000		
	Yes	1.211	1.128, 1.301	<0.001	1.201	1.039, 1.387	0.013
Pregnancy outcome history	All live births	1.000			1.000		
	>1 pregnancy a still birth/Abortion	1.202	1.068,1.354	0.002	1.082	0.895, 1.308	0.417
	No outcome reported	1.239	1.142,1.344	<0.001	1.306	1.046, 1.629	0.018
Maximum parity		1.021	1.005, 1.037	0.009	0.486	0.395, 0.597	<0.001
Area of residence	Rural	1.000			1.000		
	Urban	0.635	0.535, 0.753	<0.001	0.808	0.554, 1.178	0.269
	Peri-urban	1.281	1.189, 1.379	<0.001	1.194	1.037, 1.374	0.014
HIV status	HIV tested and negative	1.000			1.000		
	HIV tested and positive	11.425	9.348, 13.963	<0.001	7.842	6.261, 9.823	<0.001
	Never tested	5.421	4.485, 6.551	<0.001	3.275	2.615, 4.103	<0.001

External migration (whether in- or out-migration) was positively associated with all-cause mortality, and with HIV-related and all other cause-specific mortality separately with increased hazards of more than 61%. While a minimum self-reported poor health status was significantly associated with increased hazards of all-cause mortality and mortality due to HIV/TB-related causes (Tables 3–5), no significant association was found with mortality due to causes not related to HIV/TB. A positive HIV status was associated with an increased hazard of dying from all-causes and specific causes of death, with the greatest magnitude in women who died from HIV/TB-related causes (Tables 3–5). On the other hand, ever having been employed and ever been married were associated with decreased hazards of dying from all-causes as well as from HIV/TB-related causes of death (Tables 3 and 4).

**Table 4:** Risk factors associated with HIV and /or TB-related mortality, ACDIS

		Univariable analysis			Multivariable analysis		
			
Explanatory variables	Categories of explanatory variables	Crude hazards ratio	95% CI	P value	Adjusted Hazards ratio	95% CI	P value
Women age	15–19	1.000	,		1.000		
	20–24	6.127	4.705, 7.980	<0.001	2.668	1.522, 4.678	0.001
	25–29	14.863	11.545, 19.137	<0.001	4.682	2.294, 9.559	<0.001
	30–34	18.910	14.683, 24.355	<0.001	5.342	2.117, 13.481	<0.001
	35–39	16.172	12.476, 2.963	<0.001	4.114	1.294, 13.074	0.017
	40–44	13.823	10.594, 18.036	<0.001	2.362	0.582, 9.592	0.229
	45–49	14.056	10.705, 18.459	<0.001	2.416	0.469, 12.425	0.291
Self-reported minimum general health	Good	1.000			1.000		
	Fair	1.519	1.285,1.797	<0.001	3.485	1.635,7.428,	0.001
	Poor	3.675	2.857,4.729	<0.001	9.928	3.063, 32.184	<0.001
Ever employed	No	1.000			1.000		
	Yes	0.553	0.501, 0.610	<0.001	0.710	0.604, 0.836	<0.001
Ever married	No	1.000			1.000		
	Yes	0.625	0.556, 0.702	<0.001	0.482	0.387, 0.600	<0.001
Migration status	Never moved	1.000			1.000		
	Internal only	0.475	0.409, 0.553	<0.001	0.665	0.517, 0.857	0.002
	External only	1.602	1.432, 1.793	<0.001	1.711	1.363, 2.148	<0.001
	Both internal and external	0.377	0.341, 0.417	<0.001	0.541	0.448, 0.654	<0.001
Minimum household index class	Upper 20%	1.000			1.000		
	Middle 40%	1.904	1.579, 2.294	<0.001	1.425	0.967, 2.099	0.073
	Lower 40%	1.999	1.669, 2.394	<0.001	1.505	1.014, 2.234	0.042
Highest education level	More than 8 years	1.000			1.000		
	Less than 8 years	1.972	1.778, 2.187	<0.001	1.378	1.137, 1.669	0.001
	Never gone to school	1.645	1.347, 2.009	<0.001	1.372	1.000, 1.884,	0.050
Ever used contraception	No	1.000			1.000		
	Yes	1.316	1.211, 1.431	<0.001	1.314	1.099, 1.569	<0.003
Pregnancy outcome history	All live births	1.000			1.000		
	>1 pregnancy a still birth/abortion	1.124	0.976, 1.295	0.091	1.066	0.847, 1.341	0.587
	No outcome reported	1.144	1.039, 1.259	0.008	1.346	1.022,1.774	0.035
Maximum parity		1.036	1.018, 1.055	<0.001	0.505	0.394, 0.649	<0.001
Area of residence	Rural	1.000			1.000		
	Urban	0.517	0.415, 0.643	<0.001	0.733	0.438, 1.227	0.237
	Peri-urban	1.296	1.189, 1.412	<0.001	1.252	1.058, 1.482	0.009
HIV status	HIV tested and negative	1.000			1.000		
	HIV tested and positive	25.567	18.606,35.134	<0.001	17.767	12.290, 25.685	<0.001
	Never tested	10.232	7.509, 13.942	<0.001	6.49	4.479, 9.425	<0.001

**Table 5:** Risk factors associated with other causes (Combined) other than HIV and /or TB, ACDIS

		Univariable analysis			Multivariable analysis		
			
Explanatory variables	Categories of explanatory variables	Crude hazards ratio	95% CI	P value	Adjusted hazards ratio	95% CI	P value
Women age	15–19	1.000			1.000		
	20–24	2.111	1.513, 2.945	<0.001	1.761	0.965, 3.211	0.065
	25–29	3.429	2.484, 4.733	<0.001	3.649	1.974, 6.744	<0.001
	30–34	4.050	2.919, 5.620	<0.001	3.344	1.739, 6.429	<0.001
	35–39	3.644	2.576, 5.155	<0.001	3.277	1.647, 6.520	0.001
	40–44	4.318	3.063, 6.086	<0.001	1.937	0.895, 4.189	0.093
	45–49	5.388	3.824, 7.591	<0.001	2.184	0.909, 5.247	0.081
Self-reported minimum general health	Good	1.000			1.000		
	Fair	0.836	0.608, 1.149	0.269	0.564	0.399, 0.797	0.001
	Poor	1.694	0.986, 2.908	0.056	0.815	0.437, 1.520	0.521
Ever employed	No	1.000			1.000		
	Yes	0.814	0.689, 0.961	0.015	1.040	0.796, 1.360	0.769
Ever married	No	1.000			1.000		
	Yes	0.794	0.651, 0.969	0.023	0.572	0.408, 0.804	0.001
Migration status	Never moved	1.000			1.000		
	Internal only	0.405	0.306, 0.536	<0.001	0.575	0.388, 0.853	0.006
	External only	1.255	1.008, 1.568	0.045	1.621	1.121, 2.343	0.010
	Both internal and external	0.312	0.259, 0.375	<0.001	0.329	0.239, 0.453	<0.001
Minimum household index class	Upper 20%	1.000					
	Middle 40%	1.116	0.846, 1.473	0.437			
	Lower 40%	1.055	0.809, 1.375	0.690			
Highest education level	More than 8 years	1.000			1.000		
	Less than 8 years	1.636	1.351, 1.982	<0.001	1.248	0.899, 1.730	0.184
	Never gone to school	1.547	1.082, 2.211	0.017	1.364	0.812, 2.303	0.240
Ever used contraception	No	1.000					
	Yes	0.976	0.833, 1.143	0.762			
Pregnancy outcome history	All live births	1.000			1.000		
	>1 pregnancy a still birth/abortion	1.554	1.219, 1.982	0.002	1.311	0.915, 1.876	0.140
	No outcome reported	1.586	1.332, 1.888	<0.001	1.143	0.756,1.728	0.526
Maximum parity		0.985	0.949, 1.021	0.415	0.363	0.240,0.549	<0.001
Area of residence	Rural	1.000			1.000		
	Urban	0.701	0.488, 1.006	0.067	0.608	0.316,1.172	0.137
	Peri-urban	1.287	1.095, 1.513	0.025	1.215	0.939, 1.573	0.139
HIV status	HIV tested and negative	1.000			1.000		
	HIV tested and positive	3.315	2.387, 4.604	<0.001	2.590	1.790, 3.750	<0.001
	Never tested	2.399	1.822, 3.159	<0.001	1.703	1.218, 2.382	0.002

## Discussion

Overall, MR was 14.6 per 1,000 person-years in women of reproductive age in this area over the period 2000–2009. HIV/TB was the leading cause of mortality among women of reproductive age in this rural area over the period 2000–2009, and it accounted for nearly three-quarters of deaths overall (73%). The most important factors associated with all-cause and cause-specific mortality were a woman's age, migration status, HIV status, and self-reported health; the effect and/or magnitude of effect of these variables varied significantly within the different mortality groups (all-cause, HIV/TB-related causes and causes other than HIV/TB).

MRs due to HIV/TB-related causes significantly differed across the years, from as low as 5.9 per 1,000 pyo in 2009 to as high as 14.5 per 1,000 pyo in 2003. As for this study follow-up ceased at the end of 2009, there was little time to assess the impact of the public HIV Treatment and Care Program on MR at population level. More recent studies from the Africa Centre have documented an association between declining HIV/TB-related MR and the introduction of HIV treatment in public health facilities within the study area (16, 37, 38).

Results presented here confirm the findings of an earlier study (21) in this setting, when 73% of deaths in women aged 15–44 years were related to HIV over the period 2000–2004 (21). Although this study has shown that HIV-related mortality declines with the introduction and roll-out of ART over the years, a woman who was, for example, aged 20 years when she became infected and who would have died by age 35 in the absence of ART, may now with ART die by age 49 and therefore still contribute to the number of deaths within this age group. While maternal mortality reportedly dominates the mortality in women of reproductive age in sub-Saharan Africa, (1, 39, 40) in South Africa HIV-related mortality has been dominant in these women (41), due to the high HIV prevalence in South Africa and in particular in KwaZulu-Natal province (30).

Compared to women aged 15–19 years, older women had an increased risk of dying from all-causes and specific causes of death. These results confirm earlier findings from the same setting which showed that women aged 26 years and older were more likely to die of HIV/TB and injury-related causes than their younger counterparts (19, 20), which is in line with the pattern of HIV acquisition by age (42).

Women with a minimum self-reported poor health were significantly more likely to die from all-cause and/or HIV/TB-related mortality than those who reported to be in good health. This variable is based on perceived health status at the time of interview and for this study, we considered the minimum reported status over the study duration; although this may not be the best estimate of a person's health status, we have previously shown that self-reported health is a good proxy for risk of death in the subsequent 4–6 years (43).

An association between mortality and external migration has been reported previously from this setting; likely due to the findings that migrants have a higher risk of becoming HIV infected (9) and migrants have been shown to come home to die (20), similar to findings in a study carried out in the Agincourt Health and Demographic Surveillance Site (28) in which annually, the odds of dying from all-causes for external migrants was between 1.1 and 1.9 times higher than for residents. Thus, it is important that in settings with high HIV prevalence and substantial migration, this relationship is taken into account in evaluating mortality statistics and planning for health care services.

A surveillance HIV-positive status was associated with a higher risk of all-cause and cause-specific mortality than seen in surveillance HIV-negative status; the magnitude of the effect was highest in women who died from HIV/TB-related causes. These results agree with the findings from other studies elsewhere in sub-Saharan Africa (South Africa, Kenya, and Zimbabwe), confirming the impact of HIV infection on the mortality patterns observed in this study (21, 29, 44).

## Conclusion and recommendations

HIV/TB was the leading cause of death in women of reproductive age from 2000 to 2009 in this rural, high HIV-prevalence area of South Africa. Age, self-reported poor health status, external migration status, being HIV positive and unknown HIV status (among HIV surveillance participants) were the most significant risk factors associated with increased hazards of all-cause mortality and cause-specific mortality. It is likely that the roll-out of HIV treatment in this area, where HIV continues to be the main cause of death in women of reproductive age, will further reduce the risk of dying in this age group. The recently increased criteria for treatment eligibility in HIV infected adults will further contribute to this goal. To reduce the spread of HIV and other sexually transmitted diseases, increased community awareness and sensitization messages continue to be important.

## Footnotes

1 Underlying cause of death is defined by the World Health Organization (WHO) as the disease or injury that initiated the chain of events leading directly to death, or the circumstances of the accident or violence which produced the fatal injury.
